# Spatial and temporal trends of overweight/obesity and tobacco use in East Africa: subnational insights into cardiovascular disease risk factors

**DOI:** 10.1186/s12942-023-00342-7

**Published:** 2023-08-24

**Authors:** Barbara Chebet Keino, Margaret Carrel

**Affiliations:** Department of Geographical and Sustainability Sciences, University of Iowa, Iowa City, IA USA

**Keywords:** Cardiovascular risk factors, Overweight, Obesity, Tobacco use, Adaptive kernel density estimation, Sub-Saharan Africa, Demographic Health Survey

## Abstract

**Background:**

Cardiovascular disease (CVD) is increasing in Sub-Saharan Africa (SSA). Overweight/obesity and tobacco use are modifiable CVD risk factors, however literature about the spatiotemporal dynamics of these risk factors in the region at subnational or local scales is lacking. We describe the spatiotemporal trends of overweight/obesity and tobacco use at subnational levels over a 13-year period (2003 to 2016) in five East African nations.

**Methods:**

Cross-sectional, nationally representative Demographic and Health Surveys (DHS) were used to explore the subnational spatiotemporal patterns of overweight/obesity and tobacco use in Burundi, Kenya, Rwanda, Tanzania, and Uganda, five East African Community (EAC) nations with unique cultural landscapes influencing CVD risk factors. Adaptive kernel density estimation and logistic regression were used to determine the spatial distribution and change over time of CVD risk factors on a subnational and subpopulation (rural/urban) scale.

**Results:**

Subnational analysis shows that regional and national level analysis masks important trends in CVD risk factor prevalence. Overweight/obesity and tobacco use trends were not similar: overweight/obesity prevalence increased across most nations included in the study and the inverse was true for tobacco use prevalence. Urban populations in each nation were more likely to be overweight/obese than rural populations, but the magnitude of difference varied widely between nations. Spatial analysis revealed that although the prevalence of overweight/obesity increased over time in both urban and rural populations, the rate of change differed between urban and rural areas. Rural populations were more likely to use tobacco than urban populations, though the likelihood of use varied substantially between nations. Additionally, spatial analysis showed that tobacco use was not evenly distributed across the landscape: tobacco use increased in and around major cities and urban centers but declined in rural areas.

**Conclusions:**

We highlight the importance of de-homogenizing CVD risk factor research in SSA. Studies of national or regional prevalence trends mask important information about subpopulation and place-specific behavior and drivers of risk factor prevalence. Spatially explicit studies should be considered as a vital tool to understand local drivers of health, disease, and associated risk factor trends, especially in highly diverse yet low-resourced, marginalized, and often homogenized regions.

## Introduction

Cardiovascular disease (CVD) is a significant global health issue, contributing to high mortality, disability, and reduced quality of life. While high-income countries (HICs) have experienced a decline in CVD mortality since 2000, the burden of CVD is increasingly shifting to low- and middle-income countries (LMICs) [1]. In 2019, nearly 80% of global deaths attributed to CVD occurred in LMICs [2]. In LMICs, the burden of CVD and risk factors is increasing due to an ongoing epidemiological transition driven by urbanization, improved economies, cultural shifts, and nutrition transitions. However, the transition is coupled with limited access to health resources such as CVD and risk factors screening, prevention, and treatment [3–7].

CVD is a continuum, starting with behavioral risk factors, such as tobacco use and unhealthy diet choices that lead to CVD risk conditions, such as hypertension and overweight/obesity, and culminate with ischemic heart disease or stroke [8]. Effective interventions, including public health campaigns, early screening, and treatments, anywhere along the continuum can impede the progression of CVD and reduce adverse outcomes [9, 10]. Access to quality healthcare is crucial for CVD risk factor interventions, screening, and treatment, but LMICs face healthcare access challenges, particularly in rural areas where a significant proportion of the population resides [11, 12]. Although CVD is often perceived as an urban problem in LMICs, recent studies in Malawi and Kenya have highlighted high CVD risk factor prevalence in rural areas [13, 14]. While urban populations may have a higher risk of CVD risk conditions, insufficient access to screening and care in rural areas exacerbates the situation, underscoring the need for context-specific interventions [15].

The LMICs that constitute sub-Saharan Africa (SSA) bear a significant burden of global CVD morbidity and mortality, where the onset of CVD and associated mortality occur at younger ages [16, 17]. Urbanization and associated lifestyle changes, such as sedentary lifestyles and access to unhealthy food, is linked to CVD risk factors in SSA [18–20]. As SSA moves through the epidemiological transition, from communicable disease towards non-communicable disease (e.g. CVD), health and research resources are constrained by a double burden of disease [21, 22]. Quality healthcare access and delivery is limited by a lack of critical infrastructure, such as health care facilities, and an insufficient number of medical practitioners to cater to growing populations [11]. Limitations of healthcare access and delivery are more pronounced in rural areas, where 60% of SSA people reside [11, 12].

Overweight/obesity and tobacco use are significant and modifiable CVD risk factors in SSA [5, 23, 24] but there is limited literature on their prevalence and spatiotemporal patterns within the region. Current studies either focus on specific locations without allowing for comparisons across space and time or aggregate data from multiple SSA countries, missing the diverse population structures, cultures, and economic development levels that influence health outcomes [25, 26]. Moreover, there is a lack of literature on the spatial dynamics of CVD risk factors within and across SSA countries, including subnational and subpopulation differences. Understanding these dynamics is crucial for designing effective interventions, yet existing studies often report national-level prevalence without delving into subnational trends [26, 27].

This study addresses these knowledge gaps by analyzing cross-sectional nationally representative survey data from five East African countries over a 13-year period. The study employs regression and adaptive kernel density estimation methods to explore the subnational spatial and temporal patterns of overweight/obesity and tobacco use in an East African context. By going beyond administrative boundaries and creating continuous surfaces, the study captures local variations in disease distribution and trends. The five East African Community (EAC) nations—Burundi, Kenya, Rwanda, Tanzania, and Uganda—were selected to showcase the subnational and subpopulation variability in CVD risk factor trends which are often masked in national or regional trends.

The objective of this study is to describe the spatiotemporal trends of overweight/obesity and tobacco use at subnational levels over the 13-year period (2003 to 2016) by asking the following question: How does the prevalence of CVD risk factors (overweight/obesity and tobacco use) change over time (2003 to 2016), within subpopulations (rural vs. urban), and across the national landscape? By providing insights into the geographic variations of CVD risk factors observed within SSA countries, this study informs the development of context-specific and locally appropriate intervention strategies to address the burden of CVD in the region [28, 29]. Additionally, this study illustrates the application of adaptive kernel density estimation for survey data (e.g. Demographic and Health Surveys) to derive locally specific trends of health factors in resource-limited LMICs.

## Methods

### Study area

**Fig. 1:**
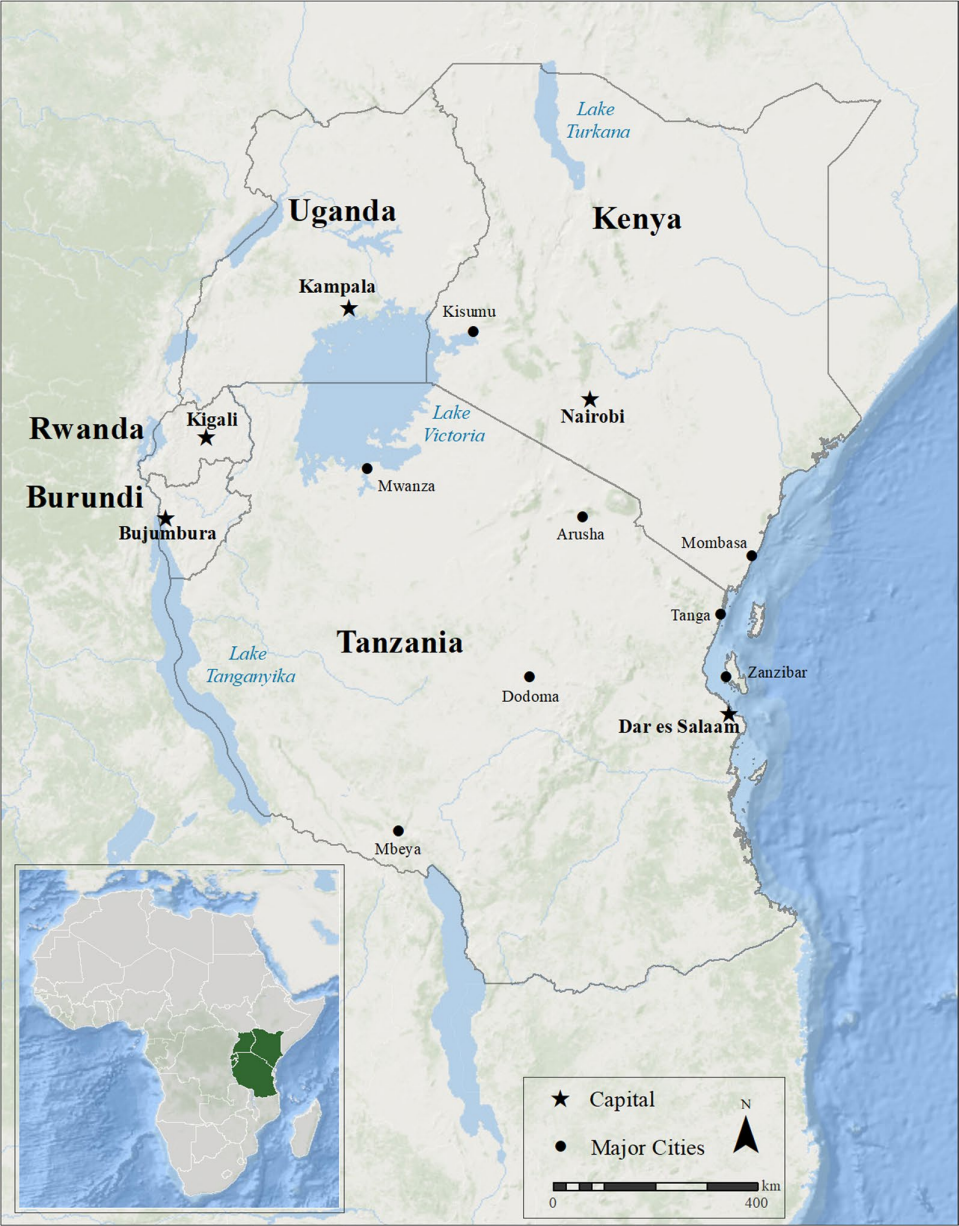
East African Community (EAC) nations included in the study

Burundi, Rwanda, Kenya, Tanzania, and Uganda are member states of the East African Community (EAC) organization in the African Great Lakes region of eastern Africa (Fig. 1). The EAC is a regional organization that links member states in economic partnership and aims to become a political federation. Member nations have unique political, economic, and disease challenges that shape the current sociocultural and health landscapes. The nations selected for this study fall into two World Bank income categories: low-income countries (Burundi, Rwanda, and Uganda) and lower-middle income countries (Tanzania and Kenya). Gross domestic product per capita (GDP) 2020 estimates in the study area were well below the world GDP (US$10,910) but varied significantly [30]. GDP ranged from US$238.99 in Burundi to US$1878.58 in Kenya [30]. Demographic and result tables are presented in order of increasing GDP (Burundi, Rwanda, Uganda, Tanzania, and Kenya) for ease of comparison and comprehension.

### Data source

Data was obtained from Demographic and Health Surveys from 2010 to 2016 in Burundi and from 2003 to 2016 in Kenya, Tanzania, Rwanda, and Uganda [31]. The Demographic and Health Survey (DHS) is a cross sectional and nationally representative survey conducted approximately every five years in low- and middle-income countries. Sampling, questionnaire, and interview techniques are spatially and temporally standardized across all surveys, allowing for comparative assessment. A stratified two-stage sample design is designed for each country using census information from national statistical boards. A weighting variable is provided with each survey to make samples nationally representative.

In most surveys since the early 2000s, DHS provides geographical coordinates (latitude and longitude) for each sample cluster. Households sampled within a cluster are assigned the same coordinates which are offset by two kilometers in urban areas and five kilometers in rural areas to maintain respondent confidentiality. Urban and rural areas are defined by national statistical boards and may vary from country to country. As there is no standard, universally accepted metric to define urban–rural areas, they may be defined by population density or infrastructure or both [32]. Data from two survey periods (2010 and 2016) in Burundi were included in this study and surveys from three survey periods (around 2005, 2010, and 2015) were included for Kenya, Rwanda, Tanzania, and Uganda. Except for the 2005 Tanzania survey, all other surveys included geographical coordinates for sample clusters.

### Variables

Overweight/obesity and tobacco use, CVD risk factors, were derived from the DHS. Interviewers collected anthropometric measures, height and weight, for women using a digital scale and measuring board. BMI data was not collected for men. Therefore, the prevalence of overweight/obesity in this study refers only to women between 15 and 49 years old who were not pregnant. Overweight and obesity categories were assigned using the World Health Organization (WHO) definition, where BMI ≥ 25 kg/m^2^ is overweight and BMI ≥ 30 kg/m^2^ is obese. To ensure enough statistical power for the analysis, overweight and obesity were combined to form one category, overweight/obese.

The DHS collects self-reported data on tobacco use habits of both sexes, therefore women between 15 and 49 years old and men between 15 and 49 years old were included in this portion of the study. Smoking and smokeless tobacco were combined into one category to ensure enough statistical power. The prevalence of tobacco use, in any form, was determined using the following DHS questions:

A) Do you currently smoke cigarettes? *(Yes or No)* in some surveys this question was asked as: Do you currently smoke cigarettes every day, somedays, or not at all? *(not at all, every day, some days)*
B) Do you currently smoke or use any other type of tobacco? *(Yes or No)* in some surveys this question was asked as: Do you currently smoke or use any other type of tobacco every day, somedays, or not at all? *(not at all, every day, some days)*
C) What other type of tobacco do you currently smoke or use? *(pipes full of tobacco, cigars or cigarillos, water pipes/shisha, snuff by mouth, snuff by nose, chewing tobacco, other)*

All affirmative answers were coded as one and summed to create an index that ranged from 0 to 3. A tobacco use variable was then created by coding respondents with a 0 score as “0” for no tobacco use and respondents with a 1 to 3 score as “1” for yes.

## Analysis

### Statistical analysis

Survey-weighted means were used to provide an estimate of the proportion of the national, rural, and urban populations with CVD risk factors (overweight/obesity or tobacco use). Each CVD risk factor was analyzed separately in each country, and results were stratified by survey period. This method allows for spatial (rural vs. urban) and temporal (survey periods) comparison of CVD risk factor prevalence within nations while accounting for the DHS complex survey design and weighting. A survey-design based Kruskal–Wallis test statistic was used to determine if CVD risk factor prevalence were significantly different across survey periods in national, rural, and urban populations within each country. All statistical analysis was performed in *R* using the *survey* package for complex survey analysis [33, 34].

### Spatial analysis

Kernel density estimation (KDE) is an interpolation method that uses features within a neighborhood (i.e., kernel) to estimate the value of raster cells [35]. Adaptive KDE uses bandwidth sizes that vary depending on the location of the sample points. Smaller bandwidths are used in heavily populated areas (i.e., urban), moderately sized bandwidths in peri-urban/suburban areas, and larger bandwidths in sparsely populated areas to capture a similar number of points for surface estimation. Adaptive KDE is most appropriate for health data that is often unevenly distributed [36–38].

The GIS data provided by the DHS links multiple individual observations to one spatial location, the sample cluster. The number of observations per sample cluster varies by location, where clusters in densely populated urban areas often have more observations than clusters in rural areas. Larmarange et al. developed a CRAN R spatial interpolation package (*prevR*) which uses Gaussian kernel estimators with an adaptive bandwidth of equal number of observations to interpolate prevalence surfaces from DHS data [39]. This method achieves high geographic detail in densely populated/sampled areas and maintains stability in sparsely populated areas with low observations. The adaptive bandwidth depends on a minimum number of observations as defined by the user. In this study, the minimum number of observations (N) was selected through the ‘Noptim()’ function in *prevR*, where the optimal N value is a function of the observed national prevalence, the number of people sampled, and the number of survey clusters [40]. The *prevR* KDE method with adaptive bandwidths has been used to map health and disease outcomes, model disease risk, equity, and social vulnerability [41–46].

In this study, KDE for survey data was used to estimate the prevalence surface of CVD risk factors (overweight/obesity and tobacco use) within nations across two survey periods (around 2010 and 2015). To illustrate the subnational change of CVD risk factor prevalence across the landscape, raster math was used to calculate a ratio of CVD risk factor prevalence between the latter survey map (around 2015) and the first survey map (around 2010). Spatial analysis was conducted in R using *prevR, sp,* and *raster* packages [34, 39, 47, 48]. Maps were created in *ESRI ArcGIS Desktop* [49].

## Results

Only women between 15 and 49 years old, who were not pregnant during the survey were included in the overweight/obesity portion of the study. A total of 182,310 women were included in the 2003 to 2016 DHS surveys. Pregnant women (n = 15,378, 8.43%) were excluded to reduce potential bias from pregnancy related weight gain, resulting in an analytical dataset of 166,932 women. The number of participants varied by country. Uganda had the lowest number of participants in this study [8] followed by Burundi (24,382), Rwanda (35,695), Kenya (44,383), and Tanzania (30,575). A total of 245,429 men and women, between age 15 and 49 years old were included in the tobacco use portion of the study. However, the number of participants varied by country. Burundi had the lowest number of participants (n = 37,170), followed by Tanzania (n = 42,410), Uganda (n = 45,331), Rwanda (n = 54,182), and Kenya (n = 66,336).

### Overweight/obesity

Overweight/obesity varied across the region and within national, urban, and rural populations of women aged 15 to 49 years in East Africa. Across all countries, there is an increasing trend in overweight/obesity prevalence over time. Generally, the prevalence appears to be higher in more recent survey periods compared to earlier ones. On a regional level, overweight/obesity prevalence was highest in Kenya (ranging from 23.17% in 2003 to 32.76% in 2014) and lowest in Burundi (ranging from 7.57% in 2010 and 7.9% in 2016) (Table 1).

**Table 1:** Survey-weighted means of overweight/obesity prevalence in women aged 15 to 49 years in East Africa, stratified by DHS survey periods and different population groups (national, urban, and rural)

Country	Survey	National		Urban		Rural	% (SE)
		*n*	% (SE)	*n*	% (SE)	*n*	
Burundi	2010	4104	7.57 (0.43)ns	909	27.45 (1.93)***	3195	5.22 (0.41)***
	2016	7908	7.9 (0.42)	1672	25.46 (1.74)	6236	5.23 (0.38)
Rwanda	2005	5211	11.54 (0.53)***	1189	19.34 (1.49)***	4022	9.96 (0.55)***
	2010	6474	16.26 (0.48)	1142	25.26 (1.19)	5332	14.65 (0.52)
	2015	6217	20.94 (0.6)	1618	37 (1.68)	4599	16.91 (0.6)
Uganda	2006	2519	16.35 (1.07)***	424	33.72 (2.87)***	2095	12.75 (1.07)***
	2011	2420	18.76 (1.04)	770	34.95 (2.14)	1650	14.29 (1.09)
	2016	5415	23.7 (0.85)	1268	34.33 (1.57)	4147	19.89 (1.02)
Tanzania	2005	9159	17.61 (0.61)***	2291	32.3 (1.45)***	6868	11.52 (0.59)***
	2010	9097	21.27 (0.69)	2398	36.01 (1.27)	6699	15.12 (0.8)
	2016	12027	28.31 (0.71)	3802	41.51 (1.25)	8225	20.68 (0.74)
Kenya	2003	7184	23.17 (0.81)***	2416	38.2 (1.43)***	4768	18.18 (0.89)***
	2009	7692	24.95 (1.18)	2410	39.63 (1.38)	5282	19.94 (1.16)
	2014	13455	32.76 (0.66)	4974	43.23 (1.14)	8481	25.81 (0.79)

Kruskal–Wallis Test Significance

*ns* not significant

^*^p < 0.05

^**^p < 0.01

^***^p < 0.001

The Kruskal–Wallis test results indicate significant differences in overweight/obesity prevalence across survey periods at a national level in Uganda, Rwanda, Tanzania, and Kenya. However, Burundi did not show significant national differences in overweight/obesity prevalence between 2010 and 2016 DHS surveys (Table 1).

There were distinct differences in overweight/obesity prevalence between urban and rural populations within each country and across all survey periods. Overweight/obesity prevalence was highest in urban areas, indicating a potential association between urbanization and overweight/obesity. Although there was no significant difference over survey periods at the national level in Burundi, overweight/obesity prevalence declined in urban areas and slightly increased in rural areas (Table 1). Rural and urban populations in all other nations experienced an increase in overweight/obesity prevalence over time (Table 1).

**Fig. 2:**
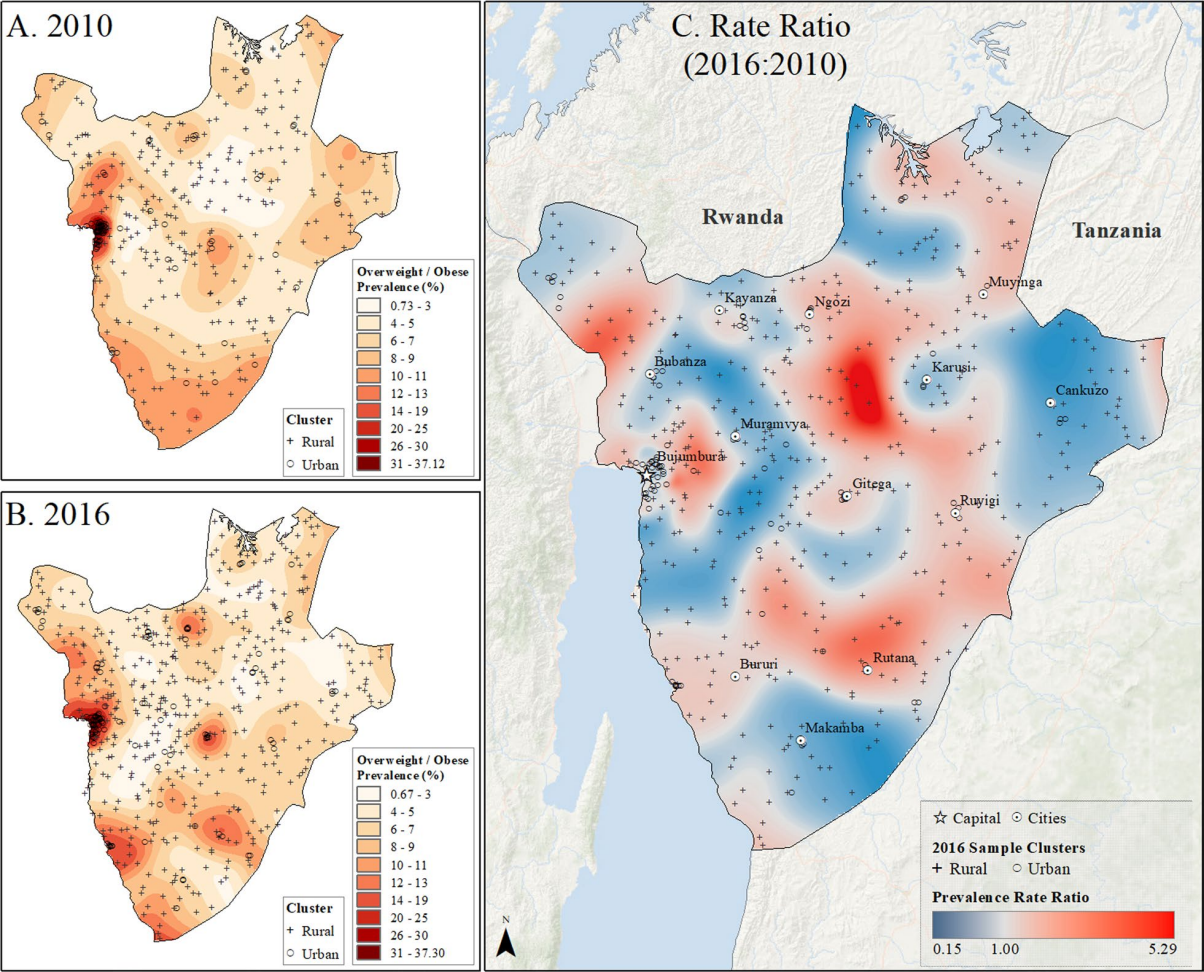
Subnational estimates of overweight/obesity prevalence for women between 15 and 49 years old in Burundi. **A** prevalence rates in 2010, **B** prevalence rates in 2016, and **C** difference ratio of prevalence between 2016 and 2010

**Fig. 3:**
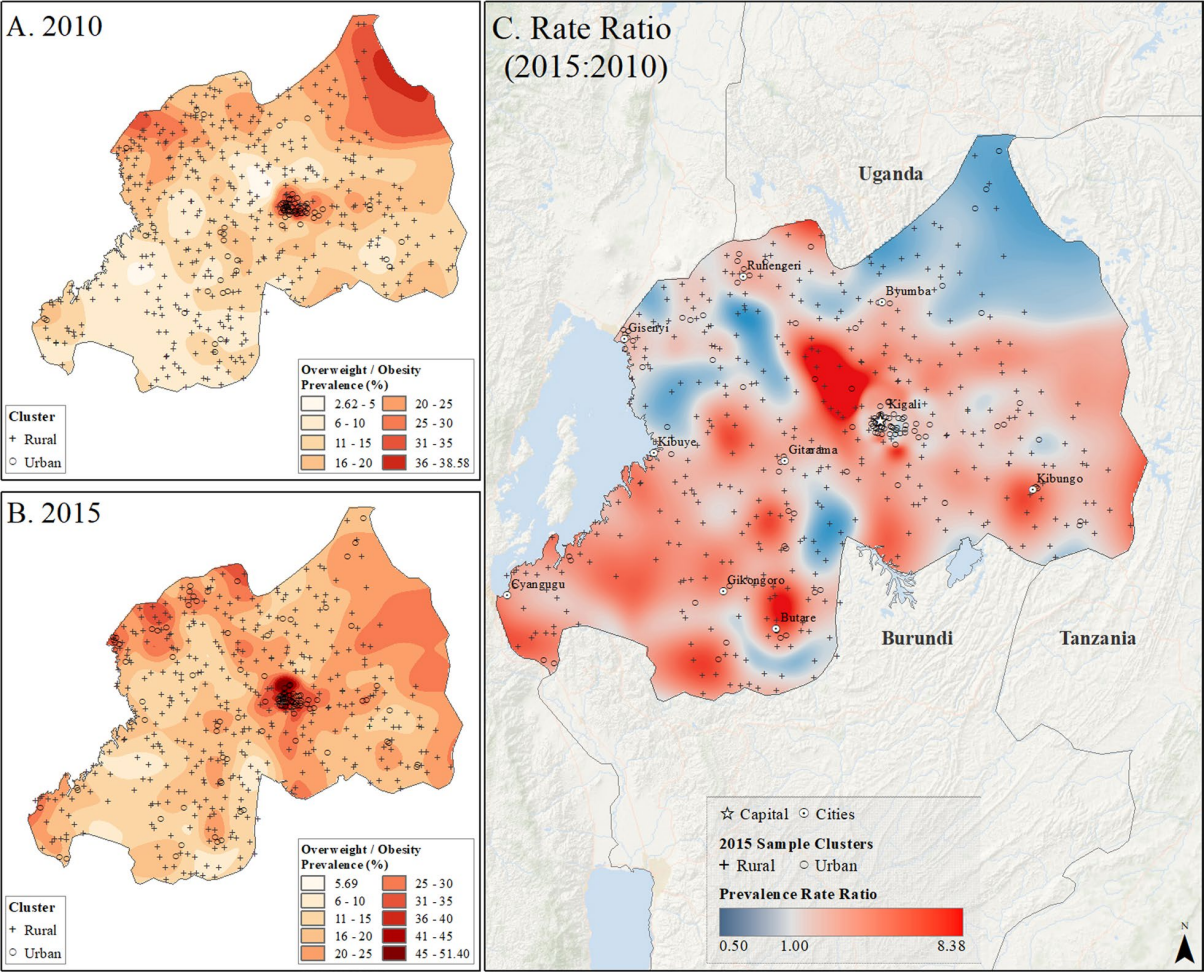
Subnational estimates of overweight/obesity prevalence for women between 15 and 49 years old in Rwanda. **A** prevalence rates in 2010, **B** prevalence rates in 2015, and **C** difference ratio of prevalence between 2015 and 2010

**Fig. 4:**
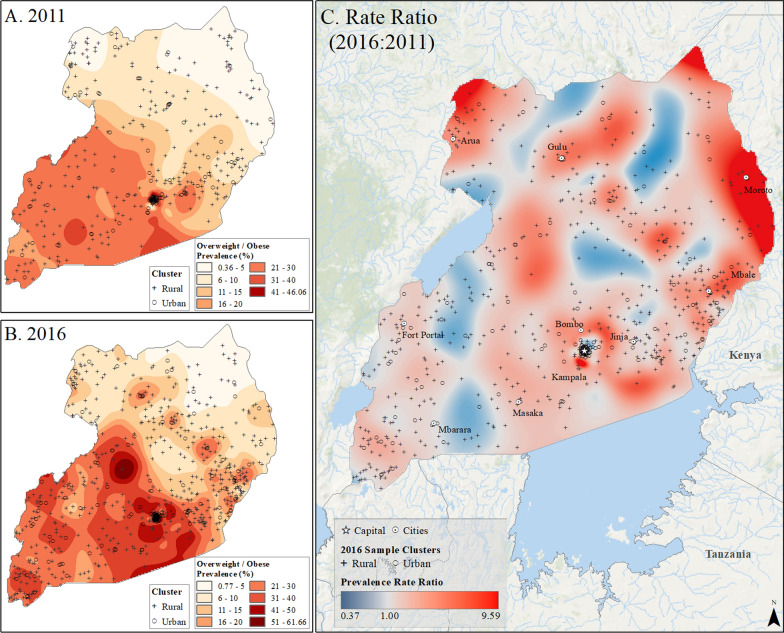
Subnational estimates of overweight/obesity prevalence for 15- to 49-year-old women in Uganda. **A** prevalence rates in 2011, **B** prevalence rates in 2016, and **C** difference ratio of prevalence between 2016 and 2011

**Fig. 5:**
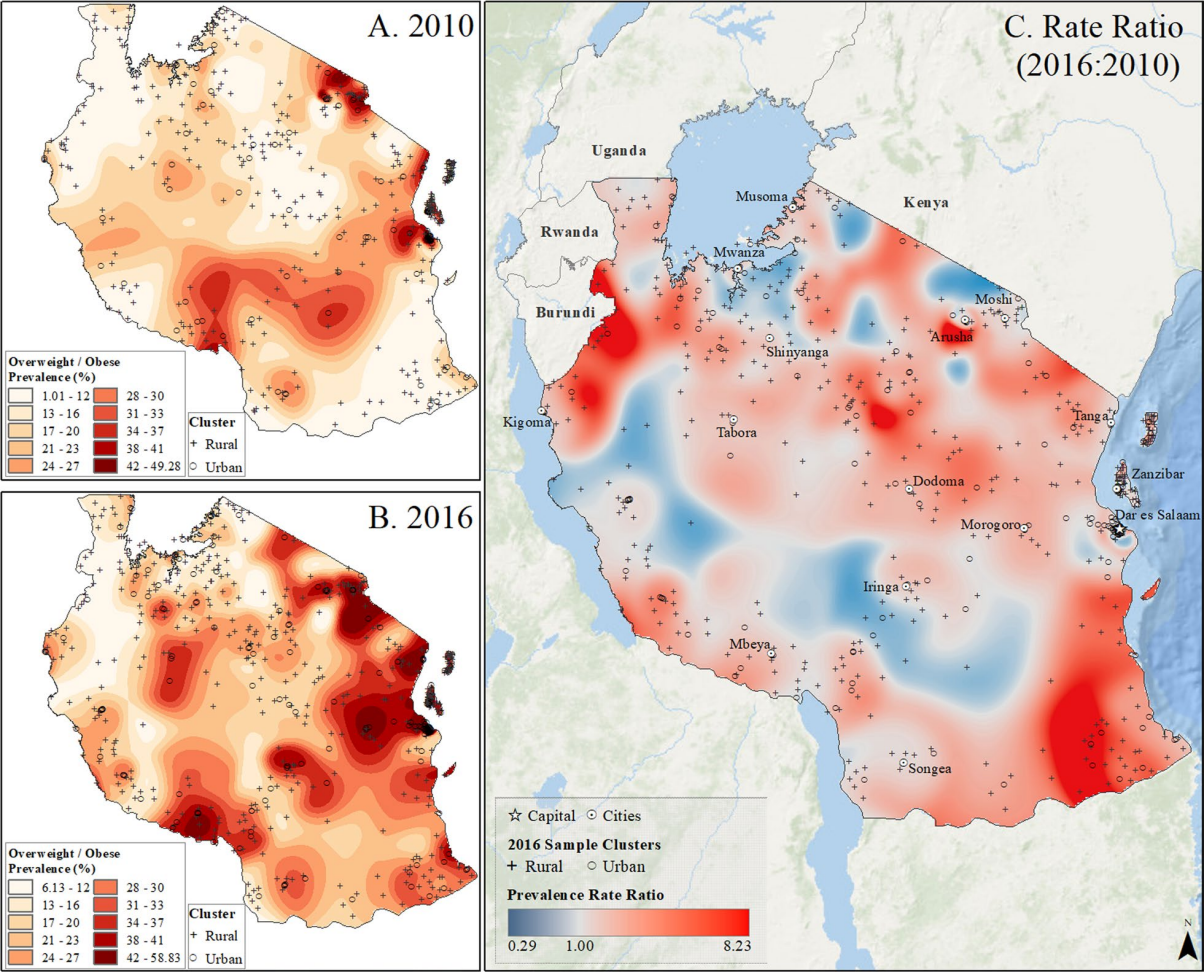
Subnational estimates of overweight/obesity prevalence for 15- to 49-year-old women in Tanzania. **A** prevalence rates in 2010, **B** prevalence rates in 2016, and **C** difference ratio of prevalence between 2016 and 2010

**Fig. 6:**
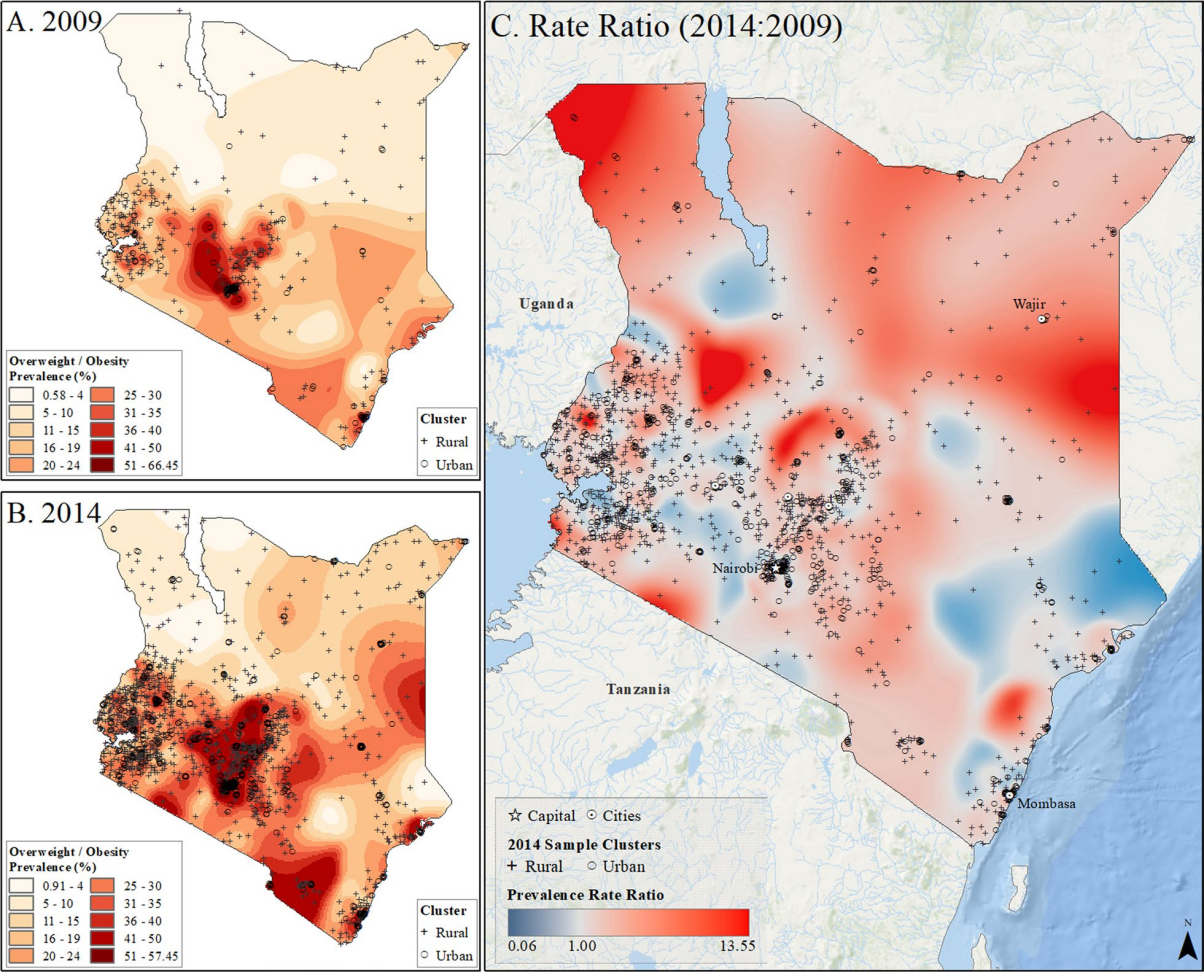
Subnational estimates of overweight/obesity prevalence for women between 15 and 49 years old in Kenya. **A** prevalence rates in 2009, **B** prevalence rates in 2014, and **C** difference ratio of prevalence between 2014 and 2009

Spatial interpolation maps (Figs. 2, 3, 4, 5 and 6) revealed the uneven distribution of overweight/obesity between urban and rural areas. Initially, capital cities had the highest rates of prevalence, a trend that persisted in the final year of study. However, there was also an increase in high prevalence rates in other urban locations. A comparison of prevalence surfaces between the first and last year of study showed an overall increase in obesity rates across the countries, particularly in rural areas (Figs. 2, 3, 4, 5 and 6).

In 2010, Burundi’s capital, Bujumbura, had the highest overweight/obesity prevalence, while by 2016, other urban locations in Burundi also reported high rates (Fig. 2). The most significant increase in overweight/obesity prevalence occurred in rural areas north of Bujumbura and near Rutana (Fig. 2). In Rwanda, Kigali and the northeastern and northwestern borders had the highest prevalence in 2010, which persisted in 2015 but with higher rates in other cities (Fig. 3). In Uganda, higher rates were observed in Kampala and southern areas in 2011, increasing across the nation by 2016, particularly in Moroto, Arua, and Kampala (Fig. 4). Tanzania showed high prevalence in Dar es Salaam, Arusha, and Moshi in 2010, which remained high in 2016, with increased rates near Arusha and the southeastern border (Fig. 5). In Kenya, Nairobi had the highest rates in 2009, which remained in central Kenya but also increased around southern border areas and the coastline in 2014 (Fig. 6).

### Tobacco use

Tobacco use prevalence across the region declined over time among adults aged 15 to 49 years (Table 2). In the last survey period (around 2015) Burundi had the highest tobacco use prevalence (7.1 ± 0.23%) while Uganda (3.45 ± 0.17%) and Tanzania (3.77 ± 0.2%) had the lowest. Tobacco use prevalence among adults aged 15 to 49 years decreased over time in all nations studied. In Burundi, for example, where data was available for 2010 and 2016, there was a 42% decline in tobacco use prevalence, from 12.31 ± 0.41% in 2010 to 7.1 ± 0.23% in 2016. Strong declines were also noted in Uganda and Tanzania during the same period (Table 2). Notably, Uganda experienced the highest relative change in tobacco use, with a decline of almost 45% over 10 years, from 7.73 ± 0.45% in 2006 to 3.45 ± 0.17% in 2016 (Table 2).

**Table 2:** Survey-weighted means of tobacco use prevalence in men and women aged 15 to 49 years in East Africa, stratified by DHS survey periods and different population groups (national, urban, and rural)

Country	Survey	National		Urban		Rural	% (SE)
		*n*	% (SE)	*n*	% (SE)	*n*	
Burundi	2010	13204	12.31 (0.41)***	3278	6.46 (0.66)***	9926	13.11 (0.46)***
	2016	23966	7.1 (0.23)	5278	4 (0.45)	18688	7.58 (0.26)
Rwanda	2005	15734	8.73 (0.29)***	3671	6.95 (0.56)***	12063	9.1 (0.33)***
	2010	19366	6.55 (0.21)	3449	5.94 (0.51)	15917	6.67 (0.23)
	2015	19082	4.52 (0.18)	4934	3.46 (0.3)	14148	4.78 (0.22)
Uganda	2006	10917	7.73 (0.45)***	1831	5.33 (1.49)***	9086	8.22 (0.46)***
	2011	10865	5.1 (0.31)	3176	2.52 (0.39)	7689	5.74 (0.37)
	2016	23549	3.45 (0.17)	5485	2.42 (0.28)	18064	3.82 (0.21)
Tanzania	2005	12964	5.66 (0.31)***	3114	4.3 (0.6)***	9850	6.2 (0.36)***
	2010	12666	5.22 (0.25)	3215	4.05 (0.42)	9451	5.68 (0.31)
	2016	16780	3.77 (0.2)	5202	3.36 (0.36)	11578	4 (0.23)
Kenya	2003	11543	8.82 (0.4)***	3836	8.32 (0.6)*	7707	8.99 (0.5)*
	2009	11700	6.41 (0.47)	3638	5.86 (0.88)	8062	6.6 (0.55)
	2014	43093	5.36 (0.17)	16262	5.35 (0.3)	26831	5.37 (0.2)

Kruskal–Wallis Test Significance

*ns* not significant

^*^p < 0.05

^**^p < 0.01

^***^p < 0.001

Across all nations and survey years, rural populations consistently had significantly higher tobacco use prevalence compared to urban populations (Table 2). The difference between rural and urban tobacco use prevalence was most evident in Burundi, 13% of the rural population used tobacco compared to 6% in urban areas. In Kenya, the nation with the highest income, the gap in tobacco use prevalence between rural and urban populations was the smallest. Similar trends of narrowing prevalence gaps between rural and urban areas were observed in Uganda and Tanzania, while Rwanda showed an increase in the difference in tobacco use prevalence between rural and urban areas over time (Table 2).

**Fig. 7:**
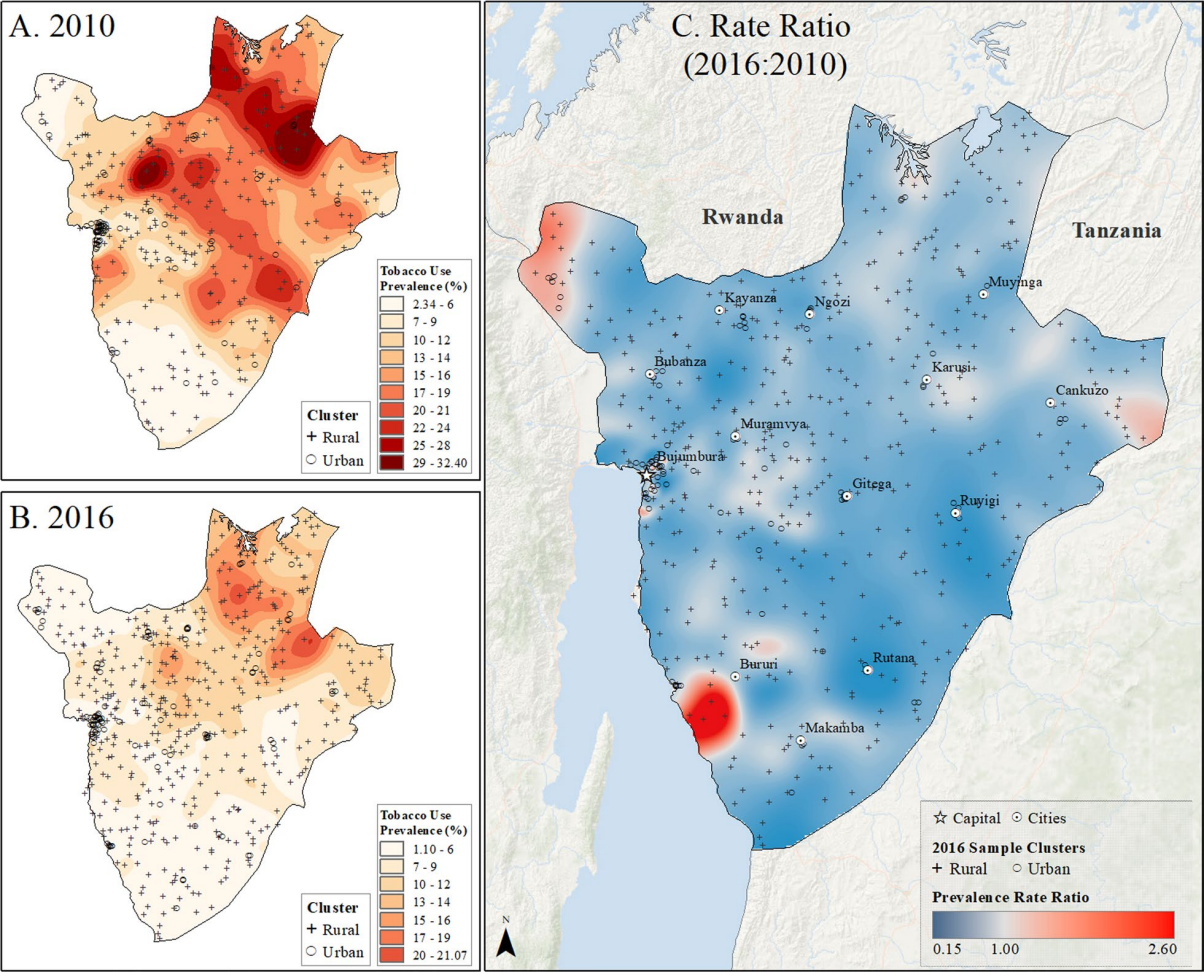
Subnational estimates of tobacco use prevalence by adults of both sexes in Burundi. **A** prevalence rates in 2010, **B** prevalence rates in 2016, and **C** difference ratio of prevalence between 2016 and 2010

**Fig. 8:**
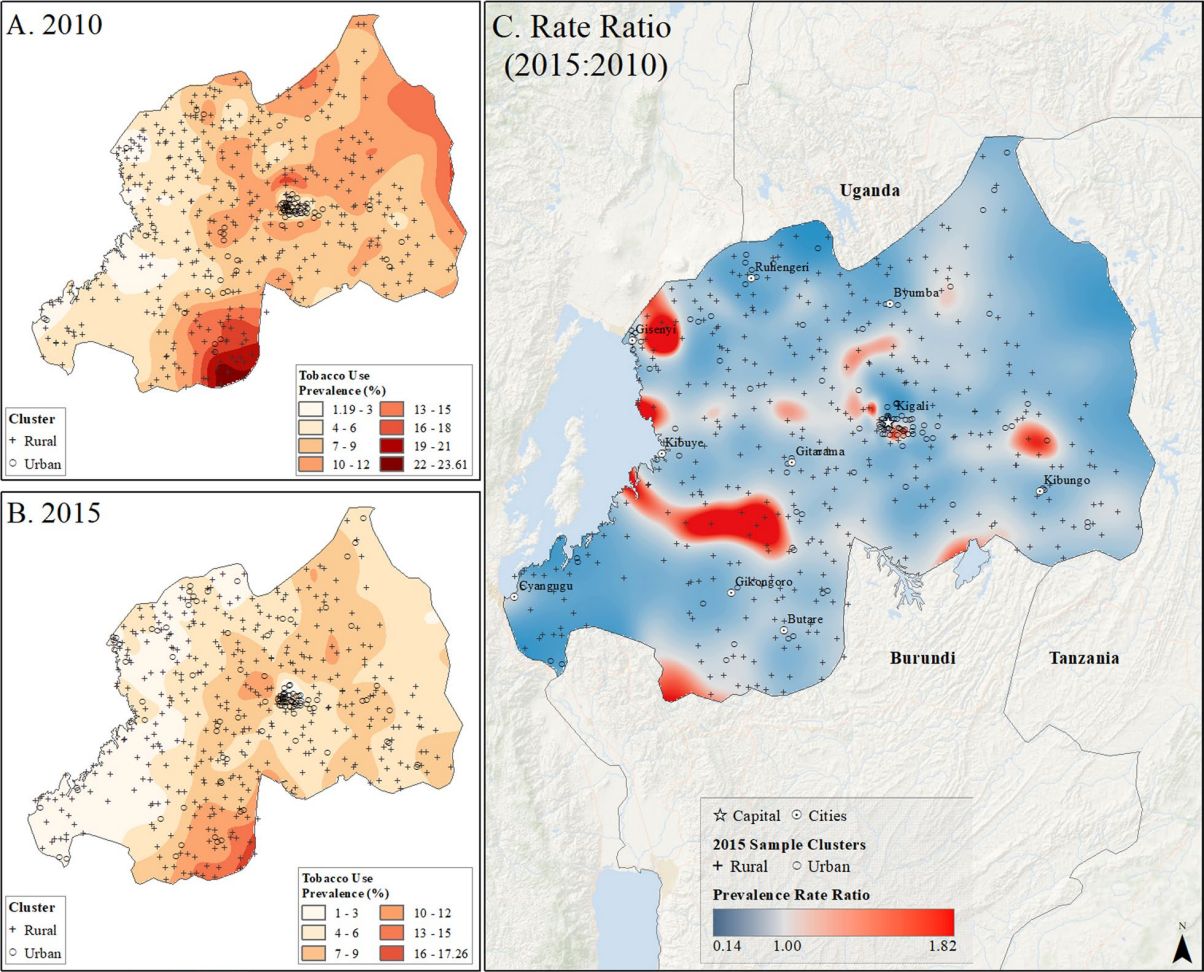
Subnational estimates of tobacco use prevalence by adults of both sexes in Rwanda. **A** prevalence rates in 2010, **B** prevalence rates in 2015, and **C** difference ratio of prevalence between 2015 and 2010

**Fig. 9:**
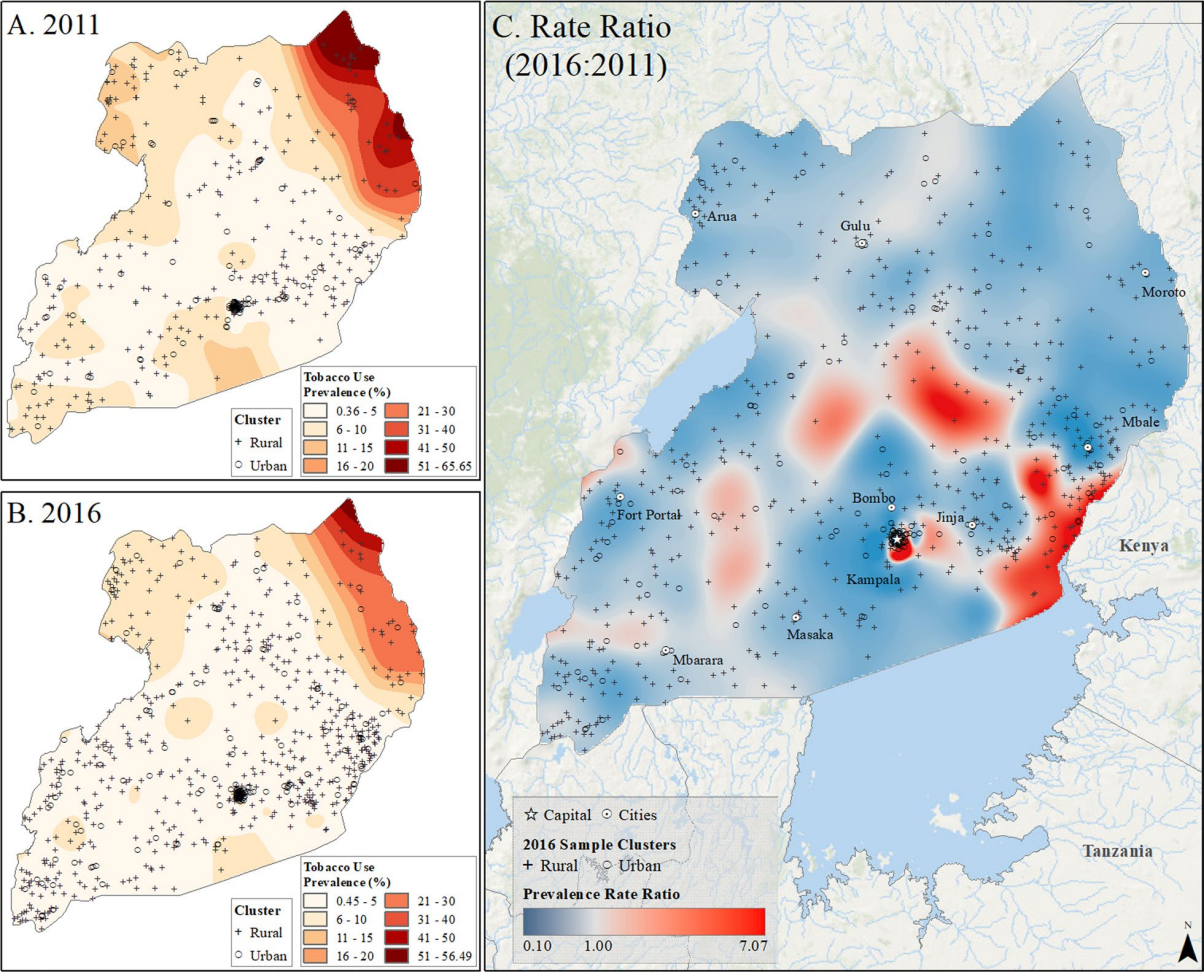
Subnational estimates of tobacco use prevalence by adults of both sexes in Uganda. **A** prevalence rates in 2011, **B** prevalence rates in 2016, and **C** difference ratio of prevalence between 2016 and 2011

**Fig. 10:**
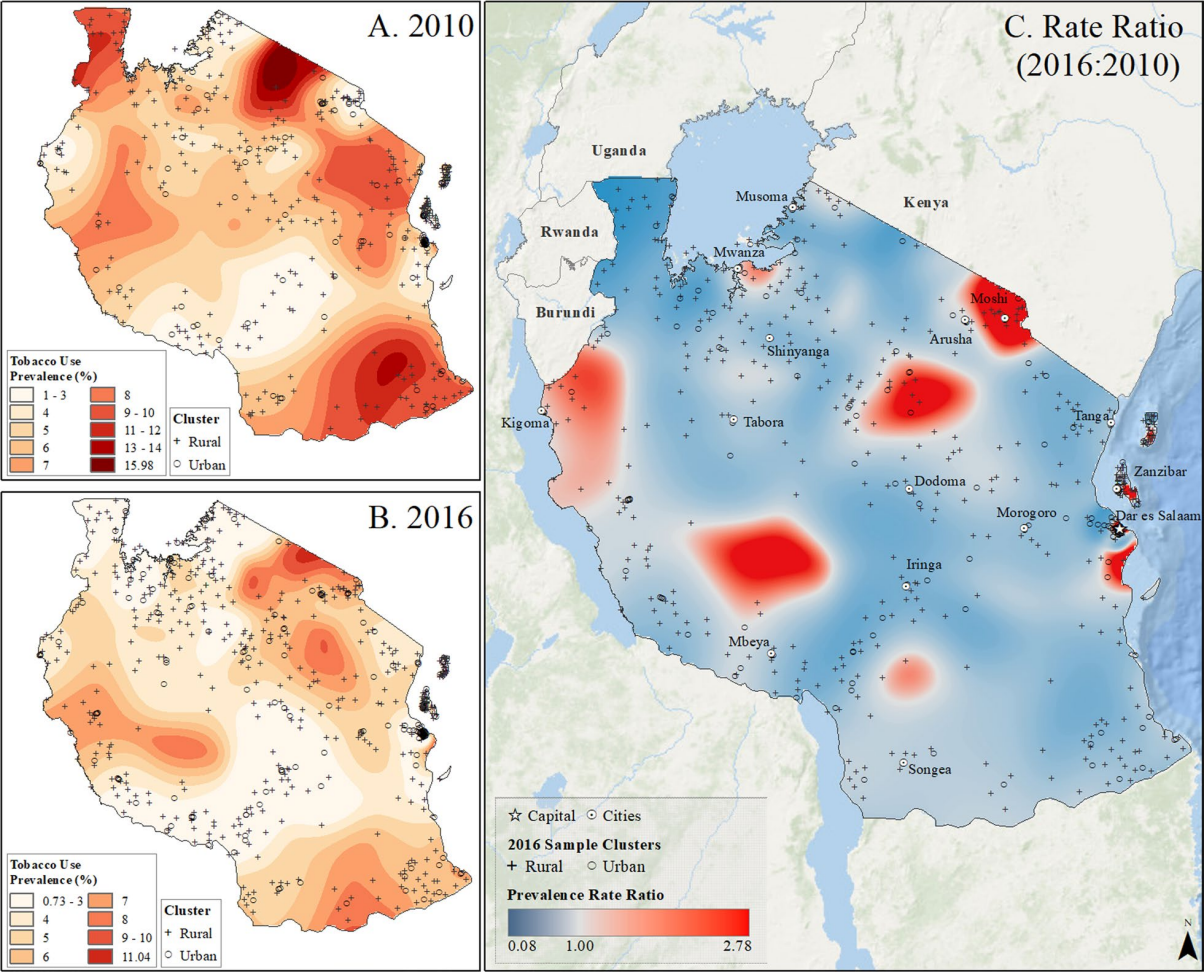
Subnational estimates of tobacco use prevalence by adults of both sexes in Tanzania. **A** prevalence rates in 2010, **B** prevalence rates in 2016, and **C** difference ratio of prevalence between 2016 and 2010

**Fig. 11:**
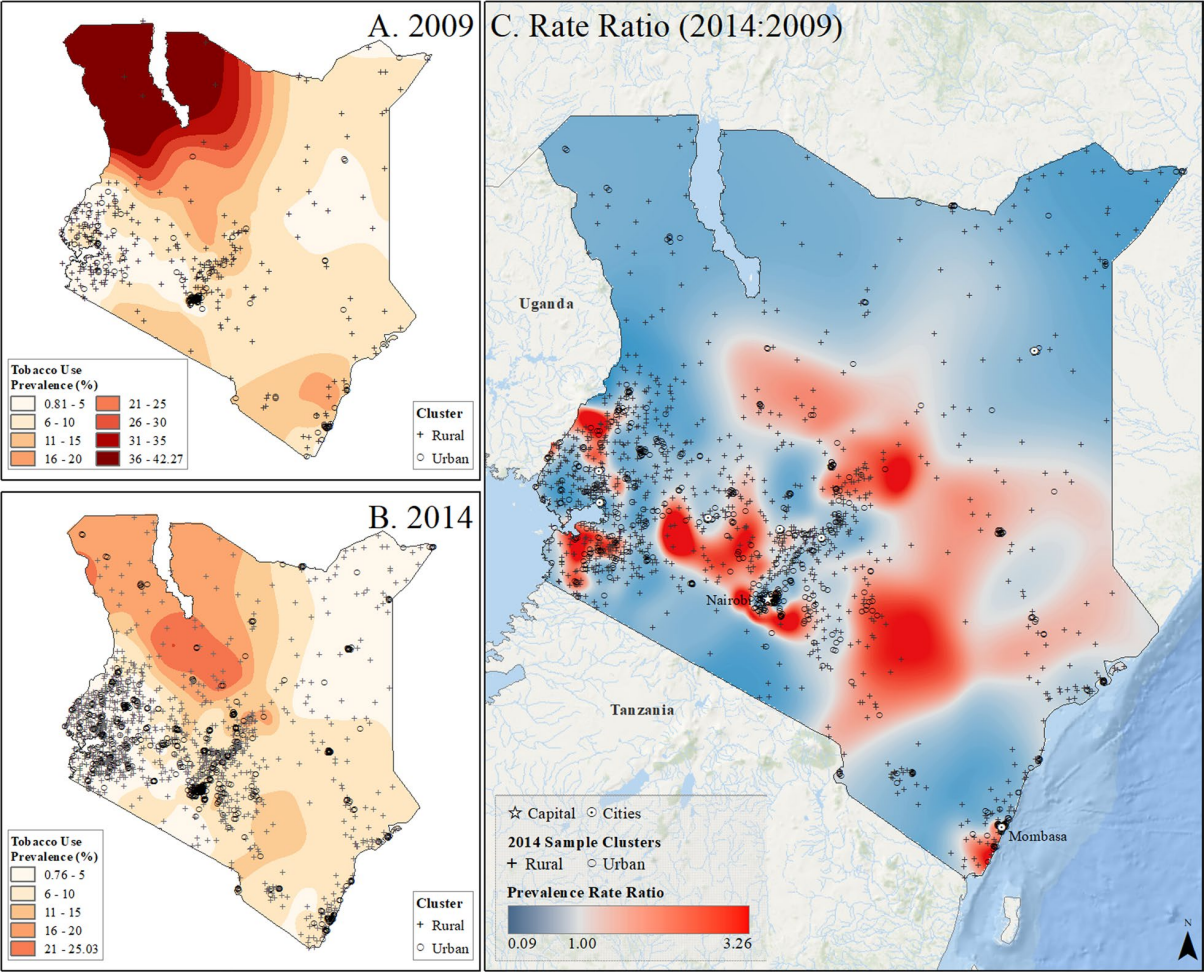
Subnational estimates of tobacco use prevalence by adults of both sexes in Kenya. **A** prevalence rates in 2009, **B** prevalence rates in 2014, and **C** difference ratio of prevalence between 2014 and 2009

The uneven distribution of tobacco use prevalence between rural and urban communities was further illustrated through prevalence surface maps (Figs. 7, 8, 9, 10 and 11). Unlike the spatial distribution of overweight/obesity prevalence, the highest prevalence of tobacco use was not located in large urban cities and was mainly located in rural areas in the first years of study. In the last year of study, overall tobacco use prevalence estimates declined over the entire landscape in all the nations (Figs. 7, 8, 9, 10 and 11). A ratio of difference between tobacco use prevalence surface estimates in the last year of study over the first year of study, showed that although the overall use of tobacco use declined in the last year of study, this decline was not evenly distributed across the landscape. In fact, tobacco use prevalence appeared to increase mainly in and around small urban centers. For example, in Burundi, tobacco use prevalence estimates in northern rural areas decreased from up to 32% in the first year of study to less than 21% in 2016 (Fig. 7). Nonetheless, some rural areas in the west and south of the country saw an increase in tobacco use over the 6-year period. Similar patterns of declining tobacco use prevalence with localized increases were observed in Rwanda, Uganda, Tanzania, and Kenya (Figs. 8, 9, 10 and 11).

In Rwanda, tobacco use prevalence in 2010 was highest on the southern border with Burundi, up to 24%, but declined to less than 17% across the country by 2015. However, pockets of increased tobacco use were observed in rural areas and on the outskirts of the capital city, Kigali (Fig. 8). In Uganda, the highest rates of tobacco use in 2011, reaching up to 66%, were found along the Kenyan border in the northeast (Fig. 9). Although tobacco use declined across the country by 2016, it increased in areas around Lake Victoria, the Kenyan border, and the capital city of Kampala (Fig. 9). Tanzania had the lowest tobacco use prevalence, ranging up to 16% in rural pockets along the northern Kenyan border and southern border in 2010. While prevalence declined to less than 11% by 2016, increased tobacco use was observed in rural areas and urban centers such as Moshi, Kigoma, Zanzibar, and Dar es Salaam (Fig. 10). In Kenya, the highest tobacco use estimates in 2009 were in the north and along the Ugandan border, up to 42% prevalence. However, by 2014, prevalence declined to less than 25% across the country, with pockets of increased tobacco use in urban and rural areas near Nairobi and along the coast between Nairobi and Mombasa (Fig. 11).

## Discussion

The objective of this study was to describe the spatiotemporal trends of CVD risk factors, overweight/obesity and tobacco use, at subnational and subpopulation levels in East Africa to highlight patterns that are often not captured in the literature that aggregates across countries, particularly LMICs. Overweight/obesity and tobacco use trends were not similar. The national prevalence of overweight/obesity increased across most nations included in the study, except Burundi, and the inverse was true for tobacco use prevalence in the region, which declined over time. Findings are in line with studies that show overweight/obesity prevalence in low-to-middle income countries (LMICs) is increasing as countries progress through stages of epidemiological and obesity transitions [50–52]. In this study, the national prevalence rate of overweight/obesity varied between nations and was associated with indicators of economic development, higher per capita gross domestic product (GDP) and urbanicity, which are also major driving forces of globalization and nutritional transition in LMICs [51, 53]. Major lifestyle and technological shifts have led to a decrease in physical activity coupled with modifications of global food systems that have increased access to cheaper, less nutritious food [54, 55].

Urban populations in each nation were more likely to be overweight/obese than rural populations, although the magnitude of overweight/obesity difference varied widely between nations. Urban populations in the lowest income nation of the group, Burundi, were almost 6 to 7 times more likely to be overweight/obese compared to rural populations. Yet in Kenya, the nation with the highest GDP in the group, urban populations were 2 to 3 times more likely to be overweight/obese compared to rural populations. Although the prevalence of overweight/obesity increased over time in both urban and rural populations, spatial analysis revealed that the rate of change was not equal across all urban and rural areas. While urban areas had higher prevalence rates at the start and end of the study, the greatest magnitude of change over time occurred in rural areas. These findings not only illustrate how analysis at broader scales, i.e., regional or national, mask variation in space and obscure the place specific indicators and drivers of overweight/obesity prevalence but may also signify obesity transition in East Africa.

Jaacks et al. , propose four stages of obesity transition as a model for the global obesity epidemic [52]. In the first stage, obesity prevalence is higher in women, people with higher socioeconomic status, and in adults more than children. Countries in south Asia and SSA, which mainly consist of LMICs, are currently in this stage. In the second stage, obesity prevalence increases in adults, the gap narrows between sexes, and socioeconomic differences among women also narrow. In the third stage obesity prevalence in lower socioeconomic groups surpasses higher socioeconomic groups and plateaus at high rates for women. Most European countries are in this stage of transition. In the final stage obesity prevalence declines in all groups, although no countries have entered this stage. Although Jaacks et al. [52] do not include urbanicity in their conceptual model, urbanicity is linked to economic development and higher socioeconomic status in LMICs [53, 56]. Therefore, the narrowing overweight/obesity gap between urban and rural areas may indicate a transition to the second stage of obesity transition in the higher income East African nations of Kenya and Tanzania.

The urban–rural overweight/obesity gap is likely linked to higher economic development in urban areas which lead to changes in employment, income, food access, physical activity, and transportation. However, as illustrated in this study and others, the rate of overweight/obesity is increasing at a higher rate in rural compared to urban areas [57]. As LMICs economies grow, rural areas adopt historically urban characteristics such as changes in infrastructure, transportation, and food access. Infrastructure changes include improved roads, access to electricity, and in some cases, improved water sources. Better roads lead to transportation shifts, such as more public transport and therefore less walking, and may also lead to changes in local food markets. With improved roads and electricity, access to cheap, highly processed obesogenic food increases coupled with more marketing efforts on radio, T.V., and print media [53, 57].

In contrast to the urban/rural patterns of overweight/obesity, rural populations were more likely to use tobacco compared to urban populations and the likelihood of use varied substantially between nations. Adults in rural Burundi were 5 to 6 times more likely to use tobacco than their urban counterparts, while rural adults in Kenya were 2 to 3 times more likely to use tobacco than urban adults. The rate of tobacco use declined in both subpopulations, however, spatial analysis showed that tobacco use was *not* decreasing across the entire landscape. Prevalence estimate maps reveal that tobacco use increased in and around major cities and urban centers in all the nations, except Burundi, but declined in rural areas.

Although the national prevalence of tobacco use declined over time in all the nations, which aligns with studies about global and national tobacco use trends, spatial analysis showed that the decline in tobacco use was not true for all places with the nations [58–60]. Current literature indicates that Sub-Saharan Africa has the lowest average rate of tobacco use compared to other WHO regions, at around 10% compared to a 22% global average in 2019 [60]. However, the region has the youngest population and most countries in SSA are in the early stages of a tobacco epidemic and tobacco use prevalence is projected to increase [61–63]. According to the WHO the number of tobacco users in SSA from 2000 to 2022 increased and is expected to continue growing [60]. All the nations included in this study are signatories of the WHO Framework Convention on Tobacco Control (WHO FCTC). The FCTC aims to reduce tobacco use through several key measures, such as: 100% smoke-free areas, health warnings labels on tobacco products, taxation, and bans on tobacco advertising, promotion, and sponsorship. However, the level of compliance varies between nations, for instance, Burundi and Uganda have implemented smoke-free environments, where all public spaces are completely smoke free, while Rwanda, Kenya, and Tanzania lack absence bans or have up to two smoke-free public spaces [59]. Egbe et al. warn that despite the recent gains in tobacco control across SSA, policy and intervention strategies are hindered transnational tobacco companies interference and are also limited by a lack of locally generated evidence [64]. This current study provides evidence at local scales that the growth of tobacco users is not evenly distributed across nations in this region and is likely happening in and around urban spaces.

Although this study was able to illustrate spatial and temporal variations in overweight/obesity and tobacco use prevalence using secondary data, there were several limitations. DHS surveys provide standardized data and large sample sizes; however, the observed trends were not longitudinal and were derived from a series of cross-section analyses of multiple surveys, which represent different sample years and sizes. Second, there are issues inherent with the definition of urbanicity in the DHS, due to a lack of a universally accepted definition of urban and rural areas, there urban and rural areas determined by national statistical offices and may differ across nations and time [32, 65]. Third, although BMI is an inexpensive and reliable anthropometric measure for large-scale surveys, it does not accurately reflect body fat composition and may result in misclassification of overweight/obesity [66]. Inherent with secondary analysis, these surveys were not designed for this study, therefore measures that are better indicators of overweight/obesity, such as waist circumference were not available. Fourth, smoking and smokeless tobacco were grouped together to create one tobacco use variable due to the questionnaire design and low prevalence rates. However, different tobacco types have users with distinct characteristics which may influence the spatial distribution and prevalence trends of each tobacco type. Fifth, self-reported tobacco use may have been underreported due to the stigma associated with tobacco use, especially among the young and women. Also, the DHS was initially designed to assess maternal, child, and nutrition health metrics and therefore women are overrepresented in the data and women past reproductive age, over 49 years old were not sampled while men up to 59 years old were sampled. As such, prevalence estimates may underestimate the true prevalence of tobacco use in older generations, particularly among older women. Lastly, the use of sample cluster data for spatial interpolation is not ideal since individual observations were linked to one spatial point and displaced by 2 to 10 km; therefore, prevalence surface estimates likely less accurate than the true prevalence distribution and variability below the cluster is masked in aggregation.

## Conclusion

To the best of the authors’ knowledge, this is the first study to explore the spatiotemporal trends of overweight/obesity and tobacco use prevalence at a subnational level in this region of SSA. Findings highlight the importance of de-homogenizing studies about CVD risk factors, such as tobacco use and overweight/obesity, in not only SSA but in other parts of the world in epidemiological transition. One question, one method, and one conclusion at regional or national levels does *not* fit all. Studies of national prevalence trends for overweight/obesity or tobacco use, which constitute most of the CVD risk factor literature in SSA, mask important information about subpopulation and place-specific behavior and drivers of risk factor prevalence. As shown in this study, although the national prevalence of tobacco use declined in all the nations, place-specific trends differed and, in some cases, increased. This type of understanding is not only vital to increase knowledge at local scales but can be a starting point for studies to understand local drivers of risk factor trends which can in turn inform policy and public health intervention.

It is imperative that as nations transition to higher levels of cardiovascular and chronic conditions, which are strongly impacted by place and culture, epidemiological analysis should occur at subnational levels and include spatial analysis, especially in regions where administrative boundaries are not static. As illustrated, spatial analysis also provides another means to understand subpopulation behavior, for instance, CVD risk factor trends in urban and rural spaces were place specific and therefore did not experience similar growth or decline patterns. Findings from this study offer insight on (1) the use of existing data, such as DHS or other national surveys, to analyze spatial trends in areas with low data resources and (2) potential future directions for research on how to utilize this type of de-homogenized analysis to build policy and create public health interventions that tailored to and appropriate for specific areas of needs.

## Data Availability

The datasets used and analyzed during the current study are available from the corresponding author on reasonable request.

## Abbreviations

CVD: Cardiovascular disease
SSA: Sub-Saharan Africa
DHS: Demographic and Health Surveys
EAC: East African Community
GDP: Gross Domestic Product
WHO: World Health Organization
BMI: Body Mass Index
INDEPTH: International Network for the Demographic Evaluation of Populations and Their Health
KDE: Kernel Density Estimation
LMIC: Low- and Middle-Income Countries
